# Hand Strength Deficit in Patients with Neurogenic Thoracic Outlet Syndrome

**DOI:** 10.3390/diagnostics11050874

**Published:** 2021-05-13

**Authors:** Alban Fouasson-Chailloux, Pauline Daley, Pierre Menu, Bastien Louguet, Guillaume Gadbled, Yves Bouju, Pierre Abraham, Marc Dauty

**Affiliations:** 1 CHU Nantes, Service de Médecine Physique et Réadapatation Locomotrice et Respiratoire, 44093 Nantes, France; pauline.daley@chu-nantes.fr (P.D.); pierre.menu@chu-nantes.fr (P.M.); marc.dauty@chu-nantes.fr (M.D.); 2 CHU Nantes, Service de Médecine du Sport, 44093 Nantes, France; bastien.louguet@chu-nantes.fr; 3 IRMS, Institut Régional de Médecine du Sport, 44093 Nantes, France; 4 Inserm, UMR 1229, RMeS, Regenerative Medicine and Skeleton, Université de Nantes, ONIRIS, F-44042 Nantes, France; 5 CHU Nantes, Clinique Chirurgicale Orthopédique et Traumatologique, 44093 Nantes, France; guillaume.gadbled@chu-nantes.fr; 6 Institut Main Atlantique, 44800 Saint Herblain, France; yves.bouju@gmail.com; 7 Sports Medicine Department, University Hospital of Angers, 49100 Angers, France; piabraham@chu-angers.fr; 8 Vascular Medicine Department, University Hospital of Angers, 49100 Angers, France; 9 Mitovasc, UMR CNRS 6015 INSERM 1083, LUNAM University, 49100 Angers, France

**Keywords:** thoracic outlet syndrome, neurogenic, strength, grip, key pinch

## Abstract

Neurogenic thoracic outlet syndrome (NTOS) is a chronic painful and disabling condition. Patients complain about upper-limb paresthesia or weakness. Weakness has been considered one of the diagnostic criteria of NTOS, but objective comparisons to healthy controls are lacking. We compared the grip and the key pinch strengths between NTOS patients and healthy controls. Grip strength was evaluated with a hydraulic hand dynamometer and the key pinch with a pinch gauge. All the patients with NTOS completed a QuickDASH. We included prospectively 85 patients with NTOS, 73% female and 27% male. The mean age was 40.4 ± 9.6. They were compared to 85 healthy subjects, 77.6% female and 22.4% male. Concerning the grip, symptomatic hands of NTOS patients had significantly 30% less strength compared to control hands (*p* ≤ 0.001), and 19% less strength compared to asymptomatic hands (*p* = 0.03). Concerning the key pinch, symptomatic hands of patients with NTOS had significantly 19.5% less strength compared to control hands (*p* ≤ 0.001). Grip and key pinch strengths had a significant correlation with the QuickDASH (r = −0.515 and r = −0.403, respectively; *p* ≤ 0.001). Patients with NTOS presented an objective hand strength deficit compared to healthy controls. This deficit was significantly correlated to the upper-limb disability. These findings confirm the interest of hand strength evaluation in the diagnostic process of patients with NTOS.

## 1. Introduction

Thoracic outlet syndrome (TOS) includes all the manifestations resulting from the compression of the upper-limb neurovascular bundle [1]. Its frequency is not clearly known and seems to be rare [2,3]. Three distinct forms are usually described according to the type of involved structures: neurogenic TOS (NTOS), venous TOS and arterial TOS [4,5]. NTOS is the most frequent form and represents more than 90% of the cases [1,4,6]. It usually associates anatomic predispositions and various local factors which may lead to intermittent compression of the brachial plexus at the supraclavicular scalene triangle and/or at the sub-coracoid space levels [6,7,8]. NTOS is a chronic painful condition responsible for functional disability and social impairment [7,9]. It affects mainly women around 40 years old in about 70% of the cases [7,10]. Usually, patients complain about upper-limb pain, paresthesia and weakness, especially during prolonged elevated arm position or during repetitive upper-limb movements. NTOS remains a challenging diagnosis due to the lack of specificity of the symptoms, the clinical examination and the radiologic exams [5,7,11,12,13]. Thereby, guidelines have recently been proposed for a more consensual diagnosis of NTOS [5,7,14,15].

Weakness is one of the main symptoms frequently reported by patients [5,7,16,17], and it has recently been proposed as one of the diagnostic criteria of NTOS [5,7,14], but objective comparisons to healthy controls are lacking [5,7,9,14]. Indeed, objective grip and pinch strength deficits remain debatable [18], and to our knowledge strength deficit of the hand in patients with thoracic outlet syndromes has not been studied in patients compared to healthy subjects. Furthermore, an increase of strength after NTOS management does not mean an initial deficit [9], because management usually implies physiotherapy and rehabilitation which include muscular strengthening [4,6]. Objective measurement of the deficit would be interesting in clinical practice both in the initial assessment as an objective diagnostic criterion of the disease, but also in the follow-up of the medical/surgical management. Indeed, there are several validated tools such as hydraulic hand dynamometers and pinch gauges, that can reliably assess strength at hand level [19,20,21].

In this study, we aimed to compare the grip and the key pinch strengths between NTOS patients and healthy control subjects. We hypothesized that patients with NTOS would have objective hand strength deficit compared to a healthy matching-population.

## 2. Materials and Methods

### 2.1. Patients

Since 2015, we have been proposing a specific protocol of rehabilitation for TOS in case of ineffective external physiotherapy and prior to a possible surgery. Patients are usually addressed by upper-limb surgeons (vascular surgeons or orthopedists), rheumatologists or vascular physicians. This protocol is usually proposed to the most disabled patients, in case of prolonged work prevention or severe quality of life impairment despite well-performed outpatient physiotherapy. In these cases, patients are potentially addressed to a surgeon in case of failure of the protocol. The rehabilitation care consists of a full-time hospitalization of 3 to 4 weeks. Before the beginning of the program, all the patients routinely perform a medical evaluation, including an upper-limb strength assessment. To be included in the study, patients had to fulfill diagnostic criteria for unilateral or bilateral NTOS according to the Consortium for Research and Education on thoracic outlet syndrome [7,14] (Table 1); they also had to accept the program of rehabilitation. Patients were excluded in case of other potential diagnosis [5].

### 2.2. Healthy Control Subjects

Healthy volunteers were recruited in the staff of the University Hospital. The criteria of exclusion were: history of neck or shoulder surgery, history of rotator cuff tendinopathy, upper-limb neurologic disorder, and high-level or elite sportspeople.

### 2.3. Grip and Key Pinch Strength Testing

The subjects were installed in a standardized position [19,20]: they were seated with their shoulder adducted and neutrally rotated, elbow flexed at 90°, with the forearm and the wrist in neutral position. Three successive trials were performed for each test. The 2 hands were evaluated in a random order in case of bilateral NTOS and for the healthy subjects, too. In case of unilateral NTOS, the non-symptomatic upper limb was tested first. Grip strength was evaluated with a hydraulic hand dynamometer (Baseline^®^, Irvington, NY, USA) and the key pinch with a pinch gauge (Baseline^®^, Irvington, NY, USA).

### 2.4. Grip and Key Pinch Strength Tests Interpretation

We took into consideration the best measure of the 3 trials for the grip strength (kg) and the key pinch (kg). The reliability previously established by Pearson product-moment correlation (PPMC) for the best grip strength trial (PPMC: 0.915 on the left side; 0.822 on the right side) and for the best key pinch trial (PPMC: 0.829; 0.748) were strong when using a Jamar Dynamometer (Asimow Engineering Co, Los Angeles, CA, USA) and a Pinch Gauge (B&L Engineering, Santa Fe Springs, CA, USA), respectively [19,22]. An excellent reliability was secondary shown on an evaluation between hydraulic hand dynamometer (Baseline^®^, Irvington, NY, USA) and Jamar Dynamometer (Asimow Engineering Co, Los Angeles, CA, USA) (ICC from 0.94 to 0.95), and between Pinch gauge (Baseline^®^, Irvington, NY, USA) and Pinch Gauge (B&L Engineering, Santa Fe Springs, CA, USA) (ICC from 0.83 to 0.91) [21].

### 2.5. QuickDash Questionnaire

All the patients with NTOS completed a French version of the QuickDASH [23], which is an 11-item-questionnaire derived from the Disability of the Arm Shoulder and Hand (DASH) questionnaire [24]. This questionnaire measures physical function and symptoms in patients with any or multiple musculo-skeletal disorders of the upper limb, and has previously been used in TOS assessment [25,26,27,28]. Participants’ responses are usually expressed in a disability score, ranging from 0 (no disability) to 100 (extreme disability).

### 2.6. Pain Assessment

Pain has been evaluated with a numeric rating scale for pain (NRS) [29]. The NRS recorded pain reported by patients from 0 (no pain) to 10 (worst pain imaginable) before strength assessment.

### 2.7. Ethics

All the participants have been recruited as part of a primary clinical study declared on clinical trials.gov with reference: NCT04145778. The protocol study was approved on 20 July 2020 by the Committee of Ethics “Comité de Protection des Personnes d’Ile-de-France II” (registration: 2019-A02787-50), and all the participants gave their verbal consent to take part into the study. According to the Committee of Ethics agreement, no written consent was needed for the participants because the study did not modify patients’ usual care; and the procedure presented minor risks for healthy volunteers from the hospital staff.

### 2.8. Statistical Analysis

The statistical analysis was performed with IBM SPSS Statistics for Windows, Version 24.0. Armonk, NY: IBM Corp. Quantitative parameters were presented as mean and standard deviation. Normality of data was tested by Kolmogorov–Smirnov test. Quantitative variable comparisons between patients with NTOS and healthy subjects were performed with independent t-tests for independent variables or with Mann–Whitney tests (if not normally distributed) and qualitative comparisons were performed with χ^2^ tests. Taking the hand as unit, we performed a Kruskal–Wallis test followed by a Dunn post hoc test to compare asymptomatic hands to symptomatic hands in patients with NTOS and to both hands of healthy controls, considering each hand independently. Spearman’s correlation coefficients (r) were calculated to assess the association between the hand strength in patients with unilateral NTOS and the QuickDASH score, the duration of the symptoms and the pain. This calculation was not possible in bilateral NTOS because only one questionnaire per patient was performed, the duration of symptoms was considered for the oldest diagnosis, and only one NRS was performed. The correlation coefficient was interpreted as followed [30]: strong correlation (r > 0.9); high (0.7 < r < 0.9); moderate (0.5 < r < 0.7), low (0.3 < r < 0.5), negligible (r < 0.3). The significant difference was determined at *p* < 0.05.

## 3. Results

### 3.1. Participants’ Characteristics

We included prospectively 85 patients fulfilling NTOS criteria (Table 1)—73% female and 27% male (Table 2). The mean age was 40.4 ± 9.6. Fifty-five patients had a unilateral NTOS (64.7%) and 30 had a bilateral form (35.3%). This group was compared to 85 healthy subjects, 77.6% female and 22.4% male. Both groups were comparable concerning age (*p* = 0.10), height (*p* = 0.10), weight (*p* = 0.42) and body mass index (*p* = 0.09). Mean duration of the NTOS symptoms was 33.5 ± 21.8 months, with no difference according to the unilateral or bilateral form of the NTOS, 32.5 ± 19.8 vs. 35.3 ± 25.2 months, respectively (*p* = 0.89). All the patients with NTOS underwent an electrodiagnostic testing which was considered abnormal for 13 patients (15%), and compatible with NTOS: two brachial plexopathies, and 11 abnormalities of the medial antebrachial cutaneous nerve conduction (out of 19 evaluations of this nerve).

The mean QuickDASH of patients with NTOS was 58.8 ± 13.4 (ranging from 31.8 to 88.6). The mean QuickDASH was not significantly different between unilateral NTOS and bilateral NTOS, 58.4 ± 14.3 vs. 59.5 ± 11.6 respectively (*p* = 0.88). At the time of the evaluation, mean NRS of pain in patients with NTOS was 5.6 ± 1.6 (ranging from 2 to 8). Mean NRS was not different between unilateral NTOS and bilateral ones, 5.6 ± 1.4 vs. 5.7 ± 1.8 (*p* = 0.59).

### 3.2. Hand Strength Assessment

The distribution of dominant and non-dominant hands was not statistically different between symptomatic, asymptomatic, and controlled hands (*p* = 0.40) (Table 3).

Concerning the grip, symptomatic hands of patients with NTOS had significantly 30% less strength compared to control hands, 25.4 ± 11.5 vs. 36.3 ± 7.9 kg, respectively (*p* ≤ 0.001), and significantly 19% less strength compared to asymptomatic hands, 25.4 ± 11.5 vs. 31.4 ± 10.5 kg, respectively (*p* = 0.03). Grip strength had 13.5% less strength on the asymptomatic side compared to controls, 31.4 ± 10.5 vs. 36.3 ± 7.9, respectively (*p* ≤ 0.001).

Concerning the key pinch, symptomatic hands of patients with NTOS had significantly 19.5% less strength compared to control hands, 7.0 ± 2.2 vs. 8.7 ± 1.7 kg, respectively (*p* ≤ 0.001). No difference was found between symptomatic and asymptomatic hands. Key pinch strength also had 11.5% less strength on the asymptomatic side compared to controls, 7.7 ± 1.9 vs. 8.7 ± 1.9, respectively (*p* ≤ 0.001).

### 3.3. Correlation between Hand Strength and Symptoms in Patients with Unilateral NTOS

Grip strength had a significant moderate inverse correlation with the QuickDASH score (r = −0.515; *p* ≤ 0.001) (Table 4). Key pinch strength had a significant low inverse correlation with the QuickDASH score (r = −0.403; *p* ≤ 0.001). Grip strength and key pinch strength were moderately correlated (r = 0.626; *p* ≤ 0.001) (Table 4). There was an absence of correlation between the NRS of pain and hand strength, both on grip and key pinch strengths (*p* = 0.06 and *p* = 0.08, respectively). No correlation was also found between the duration of symptoms and hand strength, both on grip and key pinch strength (*p* = 0.65 and *p* = 0.18, respectively).

## 4. Discussion

NTOS is a painful condition responsible for physical and mental disabilities inducing a decrease of quality of life [7]. Patients with NTOS frequently complain of hand weakness and loss of strength [5,7]. Yet, this strength deficit has never been clearly and objectively established especially compared to controls. Indeed, in spite of being a usual diagnostic criterion [5,14], it is not clear if this potential strength deficit is considered in the relation to the contralateral side or to normative data. Thus, side to side evaluation would be impossible in case of bilateral NTOS and the comparison to normative data remains debatable due to a potential variability according to the studies [31,32,33].

In this study, we assessed a group of NTOS patients whose clinical characteristics were typical of the disease. All the patients fulfilled the criteria established by the Consortium for Research and Education on thoracic outlet syndrome [7,14]. Mean age, sex-ratio and symptoms duration of our patients were also similar to those in previous studies [7,10,28] and strength measurements on the symptomatic sides of our patients were comparable to those of Ruopsa et al. recently published [34]. Electrodiagnostic testing was abnormal in 15% of our patients, certainly due to the lack of systematic evaluation of the conduction velocity in the medial antebrachial cutaneous nerve, which is reported to correlate well with a clinical diagnosis of NTOS [35].We showed that patients with NTOS had a significant strength deficit of the grip and the key pinch of 30% and 19.5% on the symptomatic sides, respectively, compared to healthy controls. We also showed less strength of 13.5% and 11.5%, respectively, on the asymptomatic side. It implies that the comparison of the symptomatic hand vs. the asymptomatic hand may undermine the importance of the deficit. These findings objectively confirmed the deficit of hand strength frequently and subjectively reported by patients with NTOS [17,36]. Interestingly, side to side comparisons showed a 19%-strength deficit of the symptomatic side compared to the asymptomatic one, but such difference was not found concerning the key pinch.

The reason for the strength deficit is certainly due to the NTOS itself, as previously shown by Braun et al. [37], who reported an increase of upper-limb strength after scalene muscle blocks. However, other explanations should be discussed. Indeed, pain condition at the time of the tests might have influenced the results due to a possible inability to correctly perform the test [38,39]. Yet, this explanation may have a low influence because we noticed no significant correlation between strength and pain at the time of the test, both on the grip and the key pinch (*p* = 0.06 and *p* = 0.08, respectively). Furthermore, pain should have influenced only the symptomatic side, yet we also found strength deficits on the asymptomatic side. However, we may evoke a link between strength reduction and the chronic painful condition due to NTOS. Indeed, in chronic low back pain, it has been shown that patients have cortical changes in the brain associated to impaired motor control of the spine muscles and consequently difficulties to exert voluntary muscle control [40]. Thus, we may hypothesize similar modifications in patients with NTOS, which could explain the strength deficit on the symptomatic side but also on the asymptomatic one. It is particularly interesting because it has been previously shown that motor training can reverse pathologic reorganization of the motor cortex in people with chronic pain [41]. Further investigations using transcranial magnetic stimulation should be performed to explore this hypothesis [42]. Another reason for the strength deficit could be a decrease of upper-limb activities which could be responsible for a strength reduction due to under solicitation. However, in such a case, we could have expected a strength reduction in relation to the duration of the pathology [43], which is not the case in this study. Taking into consideration the dominant side in our strength analyses could be discussed, because it has previously been shown that dominant hands had greater strength than non-dominant ones [32,33]. Yet, this point may have no influence on our results due to a non-significant difference of repartition dominant/non-dominant hands into the three groups (symptomatic, asymptomatic and control hands (*p* = 0.40)).

In this study, we assessed upper limb disability with the QuickDash questionnaire whose mean score was 58.8 ± 13.4 without difference between unilateral NTOS and bilateral ones (*p* = 0.88). This finding is consistent with previous studies in patients with NTOS, whose mean QuickDASH ranged from 52.9 to 62.6 [26,27,28]. We found a significant inverse correlation between the QuickDASH questionnaire and hand strength (r = −0.515 (*p* ≤ 0.001) for the grip strength and r = −0.403 (*p* < 0.01) for key pinch strength). Such correlation has previously been found in rheumatoid arthritis showing significant inverse correlation between hand strength and QuickDash but with a weaker association than in our study (r from −0.404 to −0.409 (*p* < 0.01) for the grip strength and r from -0.310 to −0.327 (*p* < 0.01) for key pinch strength) [44]. Our results confirmed a link between hand strength and upper-limb disabilities. Further studies would be interesting to assess if strength improvement is associated to a reduction of the disability. Particularly, it would be interesting to assess the efficiency of our specific rehabilitation program or other management modalities concerning strength improvement and symptoms relief. In fact, strength assessment seems very helpful to point out upper-limb weakness during the diagnostic process [14], and certainly during the follow-up to monitor the efficiency of treatments as previously shown after surgical procedure [34].

This study has several limitations. Firstly, our results could not be generalized to all the patients with NTOS because this study only concerned patients addressed to a rehabilitation center because of an ineffective external physiotherapy, with potentially a more severe form of NTOS. Secondly, despite comparable anthropometric characteristics, the patients and the controls might have been different concerning their level of activity, which could partly have explained the hand strength deficit, especially on the asymptomatic hands of patients with NTOS. Yet, we have tried to limit this bias by excluding sportspeople. Thirdly, the variability of measurements in the same individuals with NTOS has not been assessed; test–retest experiments to determine the reliability of strength evaluation would have been interesting to exactly know the error of measurement. Finally, we have only been able to assess the correlation between hand strength and, QuickDASH, pain and symptoms duration in the group of patients with unilateral NTOS, because in those with bilateral NTOS these parameters were only assessed once per patient. Yet, these parameters were comparable between these two sub-groups, which may make the results generalizable to patients with bilateral NTOS.

## 5. Conclusions

Patients with NTOS present an objective hand strength deficit on the grip and the key pinch compared to healthy controls. This strength deficit is significantly correlated to the upper-limb disability but not to the intensity of pain or to the symptoms’ duration. These findings confirm the interest of hand strength evaluation in the diagnostic process and the follow-up of patients with NTOS, especially because it is a simple, fast, and non-invasive assessment.

## Figures and Tables

**Table 1:** Characteristics of the patients with neurogenic thoracic outlet syndrome (NTOS) criteria according to the Consortium for Research and Education on thoracic outlet syndrome [7,14].

Diagnosis Criteria for NTOS	*n* (%)
No other probable diagnosis Symptoms duration ≥12 weeks	85 (100%) 85 (100%)
Principal symptoms 1a: Pain in the neck, upper back, shoulder, arm, and/or hand. 1b: Numbness, paresthesia, and/or weakness in the arm, hand, or digits.	85 (100%) 85 (100%)
Symptom characteristics 2a: Pain/paresthesia/weakness exacerbated by elevated arm positions. 2b: Pain/paresthesia/weakness exacerbated by prolonged or repetitive arm/hand use. 2c: Pain/paresthesia radiate down the arm from the supraclavicular or infra clavicular spaces.	80 (94.1%) 82 (96.5%) 71 (83.5%)
Clinical History 3a: Symptoms began after occupational, recreational, or accidental injury of the head, neck, or upper extremity, including repetitive upper extremity strain or overuse. 3b: Previous ipsilateral clavicle or first rib fracture or known cervical rib. 3c: Previous cervical spine or ipsilateral peripheral nerve surgery without sustained improvement in symptoms. 3d: Previous conservative or surgical treatment for ipsilateral TOS.	45 (52.9%) 5 (5.9%) 18 (21.1%) 80 (94.1%)
Physical examination 4a: Local tenderness on palpation over the scalene triangle and/or sub-coracoid space. 4b: Arm/hand/digit paresthesia on palpation over the scalene triangle and/or sub-coracoid space. 4c: Objectively weak handgrip, intrinsic muscles, or digit 5, or thenar/hypothenar atrophy.	81 (95.3%) 56 (65.9%) N/A *
Provocative maneuvers 5a: Positive upper limb tension test (ULTT). 5b: Positive 3-minute elevated arm stress test (EAST).	81 (95.3%) 84 (98.8%)

* Strength is the evaluated criteria of the study, and no patient exhibited overt thenar or hypothenar hand muscle atrophy on physical examination.

**Table 2:** Comparison between patients with neurogenic TOS and healthy controls.

	Patients with NTOS *n* = 85	Healthy Controls *n* = 85	*p*
Sex: Female, *n* (%) Male, *n* (%)	62 (73%) 23 (27%)	66 (77.6%) 19 (22.4%	0.48 ^a^
Age, years ± SD (min–max)	40.4 ± 9.6 (22–60)	38.2 ± 9.6 (24–67)	0.10 ^b^
Height, cm ± SD (min–max)	167.2 ± 8.9 (150–192)	169.4 ± 8.2 (155–190)	0.10 ^b^
Weight, kg ± SD (min–max)	69.0 ± 14.0 (43–105)	67.4 ± 11.9 (48–105)	0.42 ^b^
Body Mass Index, kg/m^2^ ± SD (min-max)	24.6 ± 3.9 (17.0–34.2)	23.6 ± 3.2 (17.1–31.8)	0.09 ^c^

NTOS: neurogenic thoracic outlet syndrome; SD: standard deviation. ^a^ χ^2^ test; ^b^ independent *t*-test, ^c^ Mann–Whitney test.

**Table 3:** Comparison of hand strength between symptomatic hands, asymptomatic hands in patients with NTOS and hand controls (Kruskal–Wallis test followed by Dunn post hoc test).

	Symptomatic Hands *n* = 115	Asymptomatic Hands *n* = 55	Control Hands *n* = 170	F	*p*
Dom / nonDom, *n*	63/52	33/22	85/85		0.40 ^#^
Grip strength, kg ± SD (min-max)	25.4 ± 11.5 ^a^***^, c*^ (5–54)	31.4 ± 10.5 ^b^***^, c^* (16–65)	36.3 ± 7.9 ^a^***^, b^*** (18–62)	73.97	<0.0001
Key pinch strength, kg ± SD, (min-max)	7.0 ± 2.2 ^a^*** (2.0–13.5)	7.7 ± 1.9 ^b^*** (2.5–12.5)	8.7 ± 1.7 ^a^***^, b^*** (5.5–16.0)	56.12	<0.0001

Dom: dominant hand; nonDom: non dominant hand; SD: standard deviation; ^#^ χ^2^ test for the 3 populations; ^a^ significant difference between symptomatic hands and controls; ^b^ significant difference between asymptomatic hands and controls; ^c^ significant difference between symptomatic hands and asymptomatic hands. Dunn’s test: * *p* < 0.05; *** *p* ≤ 0.001.

**Table 4:** Spearman’s correlation between hand strength and clinical factors of patients with unilateral NTOS (*n* = 55).

	QuickDASH	Pain (NRS)	Symptoms Duration	Grip Strength	Key Pinch Strength
Grip strength	−0.515 ***	−0.261 (*p* = 0.06)	−0.064 (*p* = 0.65)	1	0.626 ***
Key pinch strength	−0.403 **	−0.237 *(p* = 0.08)	−0.184 (*p* = 0.18)	0.626 ***	1
QuickDASH	1	0.422 ***	0.071 (*p* = 0.61)	−0.515 ***	−0.403 **

Spearman’s correlation: ** *p* ≤ 0.01; *** *p* ≤ 0.001.

## Data Availability

The data presented in this study are available on request from the corresponding author. The data are not publicly available due to ethical reasons.
